# Effect of Electroacupuncture on the Expression of Glycyl-tRNA Synthetase and Ultrastructure Changes in Atrophied Rat Peroneus Longus Muscle Induced by Sciatic Nerve Injection Injury

**DOI:** 10.1155/2016/7536234

**Published:** 2016-05-04

**Authors:** Meng Wang, Xiao Ming Zhang, Sheng Bo Yang

**Affiliations:** ^1^Department of Anatomy, Zunyi Medical College, Zunyi, Guizhou 563000, China; ^2^Department of Anatomy and Cell Biology, University of Kansas Medical Center, Kansas City, KS 66160, USA

## Abstract

Glycyl-tRNA synthetase (GlyRS) is one of the key enzymes involved in protein synthesis. Its mutations have been reported to cause Charcot-Marie-Tooth disease which demonstrates muscular atrophy in distal extremities, particularly manifested in peroneus muscles. In this situation, the dysfunctions of mitochondria and sarcoplasmic reticulum (SR) affect energy supply and excitation-contraction coupling of muscle fibers, therefore resulting in muscular atrophy. Although the treatment of muscular atrophy is a global urgent problem, it can be improved by electroacupuncture (EA) treatment. To investigate the mechanism underlying EA treatment improving muscular atrophy, we focused on the perspective of protein synthesis by establishing a penicillin injection-induced sciatic nerve injury model. In our model, injured rats without treatment showed decreased sciatic functional index (SFI), decreased peroneus longus muscle weight and muscle fiber cross-sectional area, aggregated mitochondria with vacuoles appearing, swollen SR, and downregulated mRNA and protein expression levels of GlyRS and myosin heavy chain IIb (MHC-IIb). The injured rats with EA treatment showed significant recovery. These results indicated that EA stimulation can alleviate peroneus longus muscular atrophy induced by iatrogenic sciatic nerve injury through promoting the recovery of GlyRS and muscle ultrastructure and increasing muscle protein synthesis.

## 1. Introduction

The sciatic nerve injury can be caused by incorrect intramuscular injection of medication in the gluteal region in clinic. This is an iatrogenic injury which can be avoided but still exists, especially in developing countries. The injury affects mostly infants [1, 2]. The injury can lead to lower limb muscle atrophy and even disability. However, the treatment of muscular atrophy is a global urgent problem because the understanding of muscle atrophy mechanisms is not completely clear. The current understanding of the mechanisms for muscle atrophy is attributed to an increase in protein degradation and/or a decrease in protein synthesis. The increase in protein degradation is mainly due to the activation of the ubiquitin-proteasome pathway, while the information on the mechanism of the protein synthesis reduction is limited [3].

A key step in protein synthesis is the covalent linkage of tRNA with corresponding amino acids catalyzed by the aminoacyl-tRNA synthetases [4]. GlyRS is one of the 20 varieties of aminoacyl-tRNA synthetases. Charcot-Marie-Tooth disease is caused by the mutations of the GlyRS gene [5]. The patients with this disease demonstrate muscular atrophy in distal extremities, particularly manifested in peroneus muscles. Nevertheless, can sciatic nerve injection injury cause the expression change of GlyRS in the peroneus muscles? Is the muscle atrophy caused by corresponding protein synthesis? It is assumed that the decrease of protein synthesis is caused by the change of GlyRS in muscle after the nerve injury, leading to muscle atrophy. However, we do not know which particular protein's synthesis is decreased. According to previous studies, the most abundant protein expression in muscle cells is myosin, which forms myofibrillar thick filaments. After nerve injury, the MHC-IIb demonstrates the most unstable feature and shows the most obvious decrease causing the fastest muscle atrophy [6–8]. Therefore, in this study, we use MHC-IIb as an index to investigate the protein expression level change in muscle atrophy.

In addition to providing energy in protein synthesis, mitochondria are also involved in cell cycle regulation and intracellular signal transduction. Insulin-like growth factor-1/phosphatidylinositol 3-kinase/protein kinase B/glycogen synthase kinase 3 beta (IGF-1/PI3K/PKB/GSK3*β*) signaling pathway is an important pathway which regulates protein synthesis. Upon activation of the pathway, PKB is transferred into mitochondria, results in GSK3*β* phosphorylation to relieve the inhibition of eukaryotic initiation factor-2B, and therefore promotes protein synthesis [9, 10]. After denervation, the mitochondria swell and become vacuolated, exhibit dysfunctions, impair protein synthesis, and aggravate muscle atrophy [11]. Although SR without ribosomes is not directly involved in protein synthesis, it regulates the excitation-contraction coupling of muscle fibers as calcium storage and thereby is closely related to muscle atrophy [12, 13].

Studies showed that electrostimulation can improve skeletal muscle atrophy through promoting axonal regeneration of injured nerve and reinnervation of muscle [14–17]. Does it affect the expression level of GlyRS and promote the recovery of ultrastructural change of peroneus longus after sciatic nerve injury? Therefore, in this study, the effect of EA on muscular atrophy and the changes of GlyRS and ultrastructure were observed. The aim of this study was to preliminarily investigate the mechanism underlying the improvement brought about by EA of rats with muscular atrophy caused by sciatic nerve injury following penicillin injection. We focus on the perspective of protein synthesis hoping to afford new thinking on the treatment of muscular atrophy.

## 2. Materials and Methods

### 2.1. Animal Care, Grouping, and Ethics Statement

One hundred and eight Sprague-Dawley (SD) rats (mean weight: 200 ± 50 g, 7–9 weeks old) were purchased from Laboratory Animal Center, Third Military Medical University (SCXK 2012-0005, Chongqing, China), either male or female randomly selected. The animals were randomly divided into 4 groups, namely, control (CON), sciatic nerve injury (SNI), CON+EA, and SNI+EA. Of these, group SNI was administrated at 1 week, 2 weeks, 4 weeks, and 6 weeks after penicillin injection-induced sciatic nerve injury, respectively; group CON+EA was administrated at 2 weeks and 4 weeks after EA stimulation of Huantiao (30th Point of Gallbladder Meridian of Foot-Shaoyang (GB30), located at the posterior upper border of the hip joint of the hind limbs, vertical needling to a depth of 6 mm) and Zusanli (36th Point of Stomach Meridian of Foot-Yangming (ST36), 5 mm below the head of the fibula under the knee joint and 2 mm lateral to the knee joint, vertical needling to a depth of 6 mm), respectively [18]; group SNI+EA was administrated at 2 weeks and 4 weeks after EA stimulation of GB30 and ST36, respectively, 2 weeks after penicillin injection of sciatic nerve injury. Totally, there were 9 animal groups, and each group had 12 rats, of which 6 were used for histological observations and the other 6 were used for molecular biological studies. The rats were housed in individual cages and fed standard rat chow and water* ad libitum*. All experiments were performed in accordance with the guidelines of the China Animal Welfare Act. This study was approved by the Animal Care and Use Committee of Zunyi Medical College. All the surgical steps were performed strictly in accordance with the principles of aseptic surgery set forth by Zunyi Medical College.

### 2.2. Sciatic Nerve Injury Induced by Penicillin Injection

Seventy-two SD rats were taken for routine disinfection of the skin, and then they were subjected to intraperitoneal anesthesia with 10% chloral hydrate at 0.3 mL/100 g. A 1.0 cm longitudinal incision was applied on the right femoral area and 0.5 cm horizontal incision under the third trochanter. The sciatic nerve was then exposed with blunt dissection, after which 200,000 U, that is, 0.5 mL, penicillin sodium (Harbin Pharmaceutical Group Co. Ltd., General Pharm. Factory, approved size: A051134107, size: 0.48 g/0.8 million units) was injected with a No. 4 needle at the outer side of the neural stem [7]. The wound was then sutured layer by layer and disinfected after surgery. Among the 72 rats that underwent surgery with penicillin injection in the sciatic nerve, 48 were selected for group SNI. Then, 12 were sacrificed at time points of 1 week, 2 weeks, 4 weeks, and 6 weeks, respectively.

### 2.3. EA Treatment

In this experiment, points of GB30 and ST36 on rats were selected as a match pair based on the principle of selecting two points on each site of the injury. Filiform needles were used to penetrate into GB30 and ST36 points. After acuesthesia, the positive pole on the G6805-II model electroacupuncture device (Qingdao Xinsheng Industrial Co., Ltd., China) was connected to GB30 point and the negative pole was connected to ST36 point with a 5 Hz output frequency using continuous wave for 10 minutes. The stimulation intensity was regulated with 2 mA and monitored by slight muscle tremor of the injured limb. EA was performed on alternate days, three times a week, and the entire course of treatment lasted 4 weeks [19]. Group CON+EA consisted of 24 normal rats receiving EA treatment without surgery and nerve injury, 12 of which received one course of treatment (group CON+EA at 2 weeks) and the other 12 received two courses of treatment (group CON+EA at 4 weeks). Of the 72 rats that were subjected to sciatic nerve injury induced by penicillin injection, 24 rats received EA at 2 weeks after the injection, 12 of which received one course of treatment (group SNI+EA at 2 weeks) while the other 12 received two courses of treatment (group SNI+EA at 4 weeks).

### 2.4. Sciatic Functional Index (SFI) Testing

**Figure 1:**
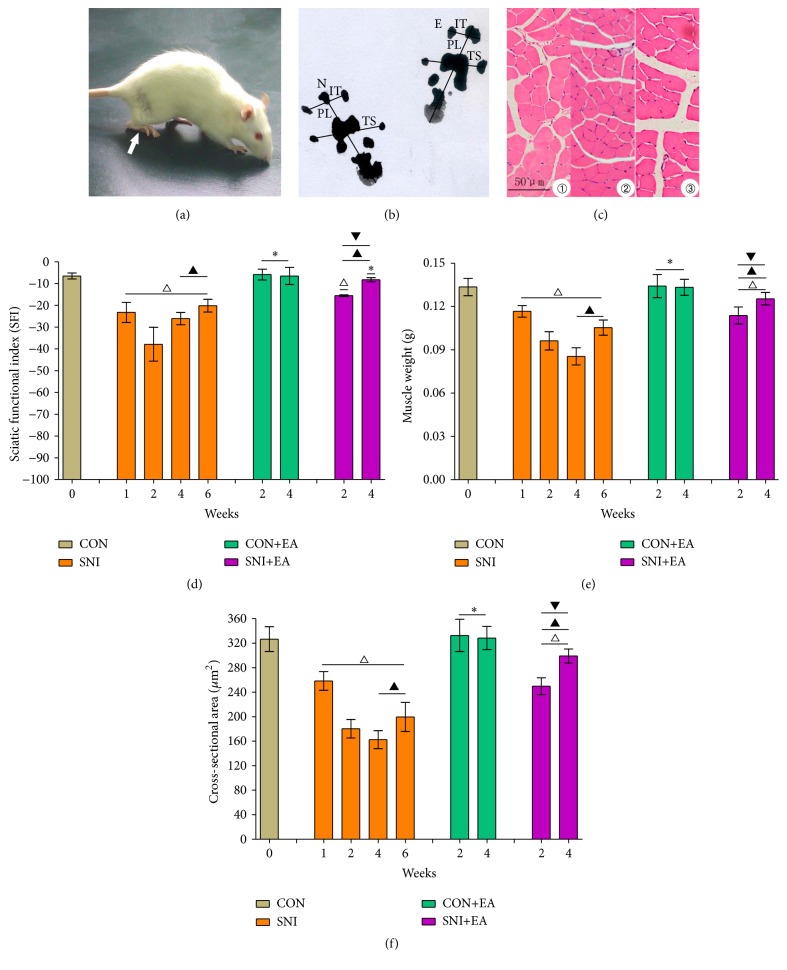
Morphology and sciatic nerve functional index (SFI) measurements. (a) Rat's clubfeet on the side operated on (indicated by the white arrow) in the group SNI at 4 weeks. (b) Rat's footprints in the group SNI at 4 weeks. N is the normal side and E is the experimental side. PL represents print length, TS represents toe spread, and IT represents intermediary toe spread. (c) An H&E-stained cross section of peroneus longus muscle from groups on a standard 50 *μ*m scale. It represents muscle cross sections from the group CON ①, the group SNI at 2 weeks ②, and the group SNI+EA at 4 weeks ③. (d–f) show SFI (d), peroneus longus muscle weight (e), and muscle fiber cross-sectional area (f) changes, respectively. ^*∗*^ *p* > 0.05 versus the group CON; ^△^ *p* < 0.05 versus the group CON; ^▲^ *p* < 0.05 versus the group SNI at 2 weeks; ^▼^ *p* < 0.05 versus the group SNI at 4 weeks or at 6 weeks.

After observing the rats' gait at every time point of each group, their soles were colored with ink and then rats were allowed to voluntarily walk from one end to the other end of the self-made footprint box, leaving 4 to 5 clear footprints of each hind leg on each side. Print lengths (PL) of normal foot (N), experimental foot (E), toe spread (TS), and intermediary toe (IT) spread were measured, respectively (Figure 1(b)). The above indexes were substituted into Bain equation to calculate the SFI, in which normal SFI = 0, while SFI = −100 indicates complete damage. Bain equation is as follows:

(1) SFI=109.5ETS−NTSNTS−38.3EPL−NPLNPL+13.3EIT−NITNIT−8.8

(see [20]).

### 2.5. Morphological Analysis

The rats were sacrificed by cervical dislocation. Peroneus longus muscle was removed quickly after observing the color and size of the muscle and was weighed and recorded. Then, partial muscle mass in venter musculi was cut into the appropriate tissue block according to the different experimental needs and was placed in formaldehyde or glutaraldehyde fixation for H&E staining or electron microscope processing. Some specimens were kept in freezer at −80°C for reverse transcriptase-polymerase chain reaction (RT-PCR) and Western blot analysis. The muscle mass for H&E staining was cross-sectioned at a thickness of 8 *μ*m. After H&E staining, the cross-sectional muscle fiber area of peroneus longus muscle was measured using the Olympus DP26 and CellSens Standard 1.11 image analysis software (Olympus Corporation, Japan). After being stained with uranyl acetate and lead citrate, the muscle ultrathin slice of the longitudinal section was observed using the Hitachi H-7650 transmission electron microscope (Hitachi Co., Ltd., Japan) and photographed.

### 2.6. RT-PCR

Total RNA was extracted from the peroneus longus of SD rats using the TRIzol reagent (Sangon Biotech Co., Ltd., Shanghai, China). One microliter of total RNA was used for reverse transcription reaction using the FastQuant RT Kit (Tiangen Biotech Co., Ltd., Beijing, China). Then, the 2x Taq PCR Master Mix kit (Tiangen Biotech Co., Ltd.) was used for PCR amplification. The PCR electrophoresis bands were photographed in the gel imager and the optical density values of the bands were measured using the Quantity One software (Bio-Rad Co., Ltd., California, USA). The optical density value of each objective gene band was divided by the optical density value of *β*-actin, which represents the mRNA expression level of the housekeeping gene. The sequences of GlyRS, MHC-IIb, and *β*-actin primers, which were designed and synthesized by Shanghai Sangon Biotech Co., Ltd., are as follows:

 GlyRS-F: 5′-GGTCAGTGTGAAGAGATTCCAG-3′.
 GlyRS-R: 5′-AAGTCAATGGTGATGCCAAAC-3′.
 MHC-IIb-F: 5′-GGCATTGAGTGGGAGTTCAT-3′.
 MHC-IIb-R: 5′-GTCTTCAACCCGGACTTCTG-3′.
 
*β*-actin-F: 5′-GAGAGGGAAATCGTGCGTGAC-3′.
 
*β*-actin-R: 5′-CATCTGCTGGAAGGTGGACA-3′.

### 2.7. Western Blot

According to the procedure instructions in the KeyGen total protein extraction kit (Nanjing KeyGen Biotech Co., Ltd., China), the muscle tissues were sheared into small pieces and were placed in a glass homogenizer. After adding the premade mixed cold lysis buffer, the glass homogenizer was manually operated 15 times, followed by 5 min centrifugation of the tissue homogenate at 10,000 rpm at 4°C. The total protein concentration of the supernatant was measured according to the instructions of bicinchoninic acid protein concentration kit (Generay Biotech Co., Ltd., Shanghai, China). Approximately 40 *μ*g of the sample was loaded in each lane for SDS-PAGE electrophoretic separation, and then the samples were transferred to a PVDF membrane. After the PVDF membrane was blocked with 5% skim milk powder, mouse anti-human GlyRS (dilution of 1 : 50) and mouse anti-chicken MHC-IIb (dilution of 1 : 2000) polyclonal antibodies (Santa Cruz Biotechnology Inc., USA) were added and incubated at 4°C overnight. Subsequently, horseradish peroxidase- (HRP-) labeled goat anti-mouse secondary antibody (Santa Cruz Biotechnology Inc., USA) was added in 1 : 2000 ratio and incubated at room temperature for 2 h. GAPDH was used as the housekeeping gene, and the film was developed using a chemiluminescent substrate. Chemiluminescence Imaging System (Bio-Rad Co., USA) was used to take photos for the bands. The optical density values of the target protein/GAPDH were measured using the Quantity One software (Bio-Rad Co., USA). The ratio represented the specific levels of protein expression.

### 2.8. Statistical Analysis

Data from this experiment were put into SPSS17.0 software package (IBM SPSS Co., USA) and then processed using one-way ANOVA with test level *α* = 0.05.

## 3. Results

### 3.1. General Observations

Compared with the group CON, the hind legs of the group SNI rats became limp and had to be dragged. One week after sciatic nerve injury, rats developed clubfeet (Figure 1(a)), the surface of peroneus longus muscles lost glossiness, and their sizes became smaller. The crus became thinner and the muscle showed significant atrophy in the group SNI at 2 weeks and 4 weeks. Compared with the group SNI at 2 weeks and 4 weeks, there was no difference under the naked eye at 6 weeks. However, no such changes were observed in the group CON+EA. The above changes showed obvious improvement in the group SNI+EA.

### 3.2. SFI Findings

The results of the SFI changes of the rats in each experimental group are shown in Figure 1(d). In the group SNI, SFI gradually reduced at 1 week and 2 weeks; and the SFI gradually recovered at 4 weeks and 6 weeks but not back to normal in comparison with the group CON (*p* < 0.05). No statistical significance was seen between the group CON+EA and the group CON. Compared with the group SNI at 2 weeks, SFI rebounded by 23.88% and 31.69% in the group SNI+EA at 2 weeks and 4 weeks, respectively; however, SFI increased by only 12.63% and 18.95% in the group SNI at 4 weeks and 6 weeks, respectively. Compared with the group SNI at 4 weeks, there was a significant difference in the group SNI+EA at 2 weeks (*p* < 0.05). Compared with the group SNI at 6 weeks, there was a significant difference in the group SNI+EA at 4 weeks (*p* < 0.05).

### 3.3. Weight Change in Peroneus Longus Muscle

The findings of peroneus longus muscle weight of SD rats in each group are shown in Figure 1(e). Compared with the group CON, the muscle weights in the group SNI were decreased by 12.62%, 27.93%, 36.02%, and 21.07% at 1 week, 2 weeks, 4 weeks, and 6 weeks, respectively (*p* < 0.05). There was the greatest decrease in the group SNI at 4 weeks. But, compared to the group SNI at 4 weeks, there was a slight recovery by 6.85% at 6 weeks (*p* < 0.05). The group CON+EA showed no difference in the muscle weight in comparison with the group CON (*p* > 0.05). The group SNI+EA at 2 weeks and 4 weeks showed increase of 13.13% and 21.84% in muscle weight compared with the group SNI at 2 weeks, respectively (*p* < 0.05). Compared with the group SNI at 4 weeks, there was a significant difference in the group SNI+EA at 2 weeks (*p* < 0.05). Compared with the group SNI at 6 weeks, there was a significant difference in the group SNI+EA at 4 weeks (*p* < 0.05).

### 3.4. Muscle Fiber Cross-Sectional Area

 Under the light microscope, the peroneus longus muscle cross section of the CON group rats was distributed as net shape, with the cytoplasm being red and the cell nucleus blue, and the muscle cross-sectional area was 326.68 ± 19.21 *μ*m^2^ (Figure 1(c)). Compared to the group CON, the peroneus longus muscle fiber in the group SNI became thinner in accordance with muscle weight, and the muscle cross-sectional areas were reduced by 20.91%, 44.81%, 50.26%, and 38.92% at 1 week, 2 weeks, 4 weeks, and 6 weeks, respectively. No statistical difference was found between the group CON+EA and the group CON. The group SNI+EA at 2 weeks and 4 weeks showed 21.27% and 36.37% recovery, respectively, compared with the group SNI at 2 weeks. Compared with the group SNI at 4 weeks, there was a significant difference in the group SNI+EA at 2 weeks (*p* < 0.05). Compared with the group SNI at 6 weeks, there was a significant difference in the group SNI+EA at 4 weeks (*p* < 0.05). These results are shown in Figures 1(c) and 1(f).

### 3.5. Ultrastructural Changes of the Peroneus Longus Muscle

In the group CON, myofibrils arranged orderly, sarcomere integrity, A band, I band, Z lines, and M lines were clearly visible; mitochondria with legible structure were distributed among the myofibrils regularly; the SR lay on myofibrils around by longitudinal distribution and were clearly visible. In the group SNI at 1 week and 2 weeks, part of myofibrils were disordered, A and I bands shortened, M lines blurred, and partial Z lines were not aligned and dissolved; the swollen mitochondria were clustered and even vacuolized; SR were gradually swollen obviously. In the group SNI at 4 weeks and 6 weeks, A and I bands were invisible, Z lines were dissolved but still leave traces, and M lines disappeared; aggregated mitochondria and vacuolization were seen in some areas; some SR were swollen and showing abnormal structures. Compared to the group CON, the ultrastructure in the group CON+EA showed no significant difference. A comparison of the group SNI+EA with the group SNI at 2 weeks showed that the abovementioned structures gradually became clear and intact. Since there was no difference in the group CON+EA at 2 weeks and 4 weeks, there was just a different change of degree in the group SNI at 1 week and 2 weeks, in the group SNI at 4 weeks and 6 weeks, and in the group SNI+EA at 2 weeks and 4 weeks. Therefore, here, we only put pictures of the group CON, the group SNI at 2 weeks, the group SNI at 6 weeks, the group CON+EA at 4 weeks, and the group SNI+EA at 4 weeks in Figure 4.

### 3.6. mRNA Expression Levels of GlyRS and MHC-IIb in Peroneus Longus Muscle

**Figure 2:**
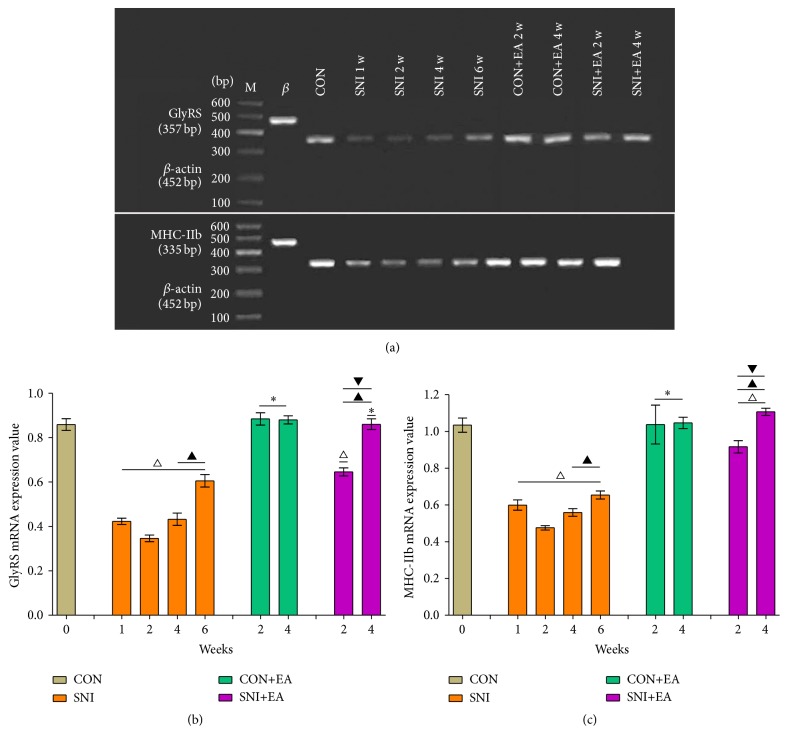
mRNA expression changes of GlyRS and MHC-IIb in peroneus longus muscle. (a) RT-PCR products of GlyRS and MHC-IIb (top to bottom). M: marker; *β*: *β*-actin; CON: control; SNI 1 w, SNI 2 w, SNI 4 w, and SNI 6 w lanes correspond to rat groups that were sacrificed at 1 week, 2 weeks, 4 weeks, and 6 weeks, respectively, after sciatic nerve injury following penicillin injection; CON+EA 2 w and CON+EA 4 w lanes correspond to normal rats who received EA after 2 weeks and 4 weeks, respectively; SNI+EA 2 w and SNI+EA 4 w lanes correspond to rats that received EA after 2 weeks and 4 weeks, respectively, 2 weeks after sciatic nerve injury. (b) and (c) are the relative mRNA expression levels of GlyRS and MHC-IIb in peroneus longus muscle in each experimental group, respectively. ^*∗*^ *p* > 0.05 versus the group CON; ^△^ *p* < 0.05 versus the group CON; ^▲^ *p* < 0.05 versus the group SNI at 2 weeks; ^▼^ *p* < 0.05 versus the group SNI at 4 weeks or at 6 weeks.

The mRNA expression changes of GlyRS and MHC-IIb in each experimental group are shown in Figures 2(a)–2(c). Compared with the group CON, the optical density value of mRNA expression of GlyRS in the group SNI was decreased by 50.77%, 59.72%, 50.84%, and 29.51% at 1 week, 2 weeks, 4 weeks, and 6 weeks, respectively (*p* < 0.05). No statistical difference was found between the group CON+EA and the group CON. Compared with the group SNI at 2 weeks, the group SNI at 4 weeks and 6 weeks showed that GlyRS expression only increased by 8.88% and 30.21%, respectively (*p* < 0.05). In the group SNI+EA at 2 weeks and 4 weeks, GlyRS expression levels increased by 34.86% and 59.86%, respectively, compared to the group SNI at 2 weeks (*p* < 0.05). The mRNA expression level of GlyRS of the group SNI+EA at 2 weeks was significantly different from that of the group SNI at 4 weeks (*p* < 0.05). The expression level of GlyRS of the group SNI+EA at 4 weeks was significantly different from the group SNI at 6 weeks (*p* < 0.05). The expression variation pattern of MHC-IIb mRNA was the same as that of GlyRS with merely slight differences in the expression values.

### 3.7. GlyRS and MHC-IIb Protein Expression Changes in Peroneus Longus Muscle

**Figure 3:**
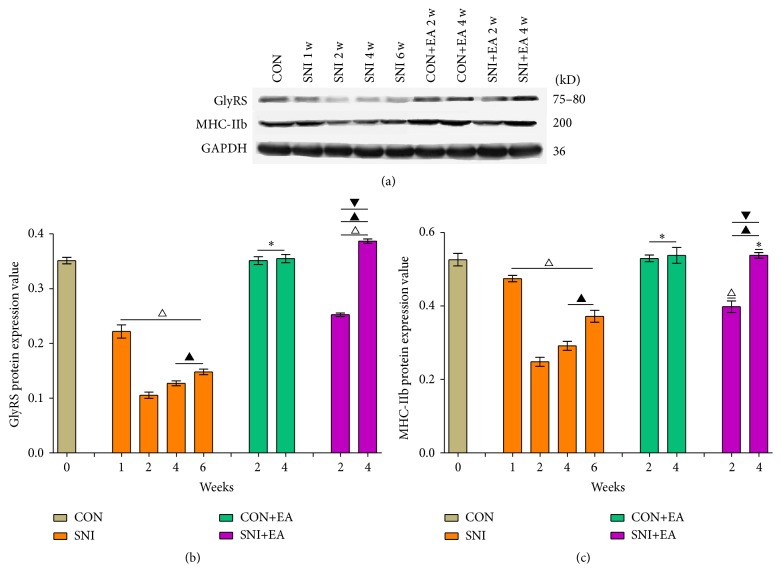
Protein expression changes of GlyRS and MHC-IIb in peroneus longus muscles. (a) Western blot results of GlyRS, MHC-IIb, and housekeeping gene (top to bottom). (b) and (c) are the specific protein expression levels of GlyRS and MHC-IIb in peroneus longus muscle in each experimental group, respectively. ^*∗*^ *p* > 0.05 versus the group CON; ^△^ *p* < 0.05 versus the group CON; ^▲^ *p* < 0.05 versus the group SNI at 2 weeks; ^▼^ *p* < 0.05 versus the group SNI at 4 weeks or at 6 weeks.

Protein expression levels are shown in Figures 3(a)–3(c). The variation patterns in the protein expression levels of GlyRS and MHC-IIb in peroneus longus muscles were the same as their mRNA expression variation patterns, with differences only in the expression degree.

## 4. Discussion 

Gluteal region injection, even with normal saline, may end up with direct sciatic nerve damage or injury by regional compression. The consequence is muscular atrophy or even disability. This brings about harm and even disaster to the patient and family [21]. Therefore, the study of the mechanism and the treatment of this muscle atrophy have been a popular concern. Although the mechanism study of an increase in protein degradation had achieved great progress in skeletal muscle atrophy caused by nerve injury [22], studies on the reduction of protein synthesis in muscle atrophy are poorly understood. At present, there are a lot of reports on the treatment of muscle atrophy, especially the treatment using acupuncture. They all have obvious improvement in muscle atrophy, but the mechanism of muscle atrophy is unknown [23, 24]. The probability of common peroneal nerve injury is higher than that of the tibial nerve because of the lateral location of the former within the sciatic nerve starting from the gluteal region. This feature makes the peroneal nerve more vulnerable for gluteal injection injury. Thus, in this study, we establish SD rat models with sciatic nerve injury-induced peroneus longus muscular atrophy by intentionally injecting penicillin, which is extremely neurotoxic, into the outer side of sciatic nerve stem and then observe the improvement effect of muscle atrophy by EA. The expression level of GlyRS and the ultrastructural changes were detected before and after nerve injury or acupuncture treatment. We tried to look into the mechanism underlying the improvement brought about by EA from the perspective of protein synthesis.

Muscle weight and muscle fiber cross-sectional area can reflect the extent of skeletal muscular atrophy [7, 25]. The SFI is an indicator of evaluating the sciatic nerve function [26]. The motor function of the mice was significantly improved by the treatment of EA after spinal cord transection [15]. Our results showed that, 1 week after sciatic nerve injection injury, the rats developed clubfeet and their hind legs became limp and had to be dragged. The size of peroneus longus muscle became smaller, muscle weight and muscle fiber cross-sectional area decreased, and SFI decreased significantly. Moreover, the muscle weight and muscle fiber cross-sectional area further reduced in the group SNI at 2 weeks. These results suggested that our muscular atrophy model of sciatic nerve injury induced by penicillin injection was successful. SFI showed the most significant decline in 2 weeks after sciatic nerve injury. The reason for this declination might be the acute nerve edema or degeneration caused by neurotoxic effect of penicillin and/or local compression to the nerve stem. The SFI shows gradual restoration in the group SNI at 4 weeks and 6 weeks. This may be due to the gradual absorption of penicillin and its toxicity, release of local pressure, and nerves regeneration.

The main component of the skeletal muscle is protein, and skeletal muscle atrophy depends on the balance of protein degradation and synthesis. However, there are many factors affecting protein synthesis. One of them, GlyRS, is an essential enzyme acylating tRNA^Gly^ with glycine involved in protein synthesis [27]. Protein synthesis is impaired when the aminoacylation of GlyRS is inhibited or damaged. Currently, people understand more about the Charcot-Marie-Tooth disease type 2D. The disease is a monogenic disease caused by GlyRS gene mutations and involves peripheral nervous system degeneration [28]. One study found that the disease has impaired protein translation in motor and sensory neurons resulting in muscle atrophy and is unrelated to impaired aminoacylation of GlyRS [29]. Our results show that the expression levels of GlyRS and MHC-IIb were gradually reduced at 1 week and 2 weeks after sciatic nerve injury, and the muscle weight, muscle fiber cross-sectional area, and the expression level of MHC-IIb were also decreased, suggesting that the sciatic nerve injury of penicillin injection had an influence on aminoacylation of GlyRS. However, further studies are needed to investigate whether the nerve injury is directly related to GlyRS. One possibility is that the reduction of MHC-IIb expression is due to an increase in protein degradation, not a decrease in protein synthesis. In group SNI+EA, the expression levels of GlyRS and MHC-IIb, the muscle weight, muscle fiber cross-sectional area, and SFI showed obvious recovery compared with the group SNI at 2 weeks, while no statistical difference was shown in the group CON+EA at 2 weeks and 4 weeks. These results indicate that the muscle atrophy can be improved by acupuncture treatment and the possible mechanism is that acupuncture restored the aminoacylation function of GlyRS in protein synthesis by promoting its expression after nerve injury except for the normal individuals. Compared with the group SNI at 2 weeks, the mild recoveries of the group SNI at 4 weeks and 6 weeks might be due to the collaborative recovery function of SD rats' own nerve regeneration ability.

**Figure 4:**
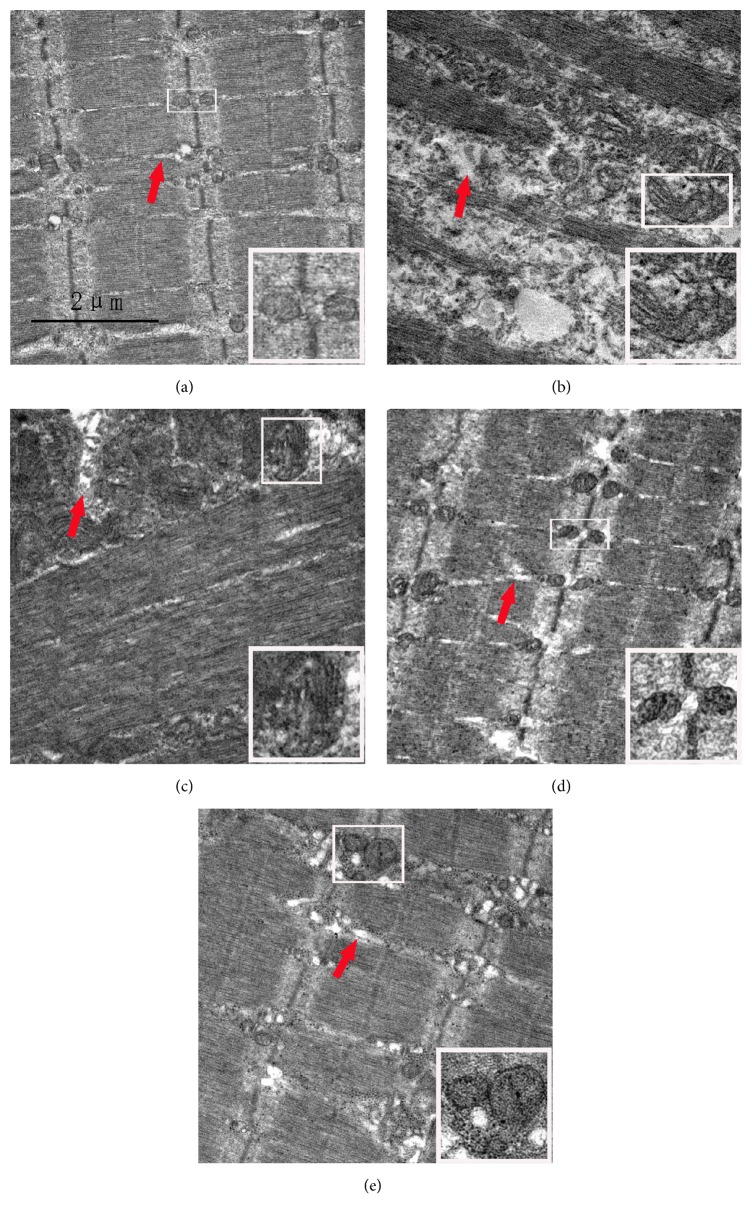
Ultrastructural changes of the peroneus longus muscle after injection-induced sciatic nerve injury and EA treatment. Bar = 2 *μ*m. (a) is the group CON; mitochondria with normal morphology (white boxes) are distributed among the myofibrils regularly; the sarcoplasmic reticula (SR) (red arrow) are clearly visible around myofibrils with longitudinal distribution. (b) and (c) are the group SNI at 2 weeks and 6 weeks; mitochondria (white boxes) and SR (red arrow) are swollen (b); aggregated and swollen mitochondria (white boxes) are seen in some areas; some SR show abnormal structures (red arrow) (c). (d) is the group CON+EA at 4 weeks; ultrastructures are similar to normal. (e) is the group SNI+EA at 4 weeks; the structures of mitochondria (white boxes) and SR (red arrow) are almost normal and distributed regularly.

On the other hand, the processing, modification, and transport of protein synthesis are involved in endoplasmic reticulum, Golgi apparatus, and mitochondria with protein synthesis mainly carried out in the free ribosomes of cytoplasm [30]. Although SR without ribosomes in skeletal muscle is not directly involved in protein synthesis, it regulates the excitation-contraction coupling of muscle fibers as calcium store, and, thereby, it is closely related to muscle atrophy. The abundant mitochondria in muscle cells can provide lots of energy for the contraction of skeletal muscle. In addition, the mitochondria are also involved in the regulation of cell cycle and cell signal transduction. Sakakima et al. put the sciatic nerves in a frozen medium and thawed them a few times by liquid nitrogen and then observed the ultrastructural changes of soleus muscle with time. At 1 day after freeze-thaw cycles, myofibrils with their Z lines not in order were only partially observed. At 3 days and 1 week, disorganized sarcomeres were gradually increased in number and size with time. At 2 weeks, the range of these disorganized sarcomeres sequentially increased, Z lines were not disrupted but highly wavy, A and I bands were not discernible, and mitochondria were clustered and enlarged. At 3 weeks, the range of these disorganized sarcomeres began to decrease and Z lines were disrupted in many sarcomeres. At 4, 5, and 6 weeks, these abnormal conditions were gradually reduced and enlarged mitochondria were seen in clusters occasionally [31]. Our results are shown in Figure 4; the ultrastructural changes in our study are similar to the above research, only with different observation time points. These results imply that the acupuncture treatment can promote the recovery of mitochondria after sciatic nerve injury, thereby restoring the functions of mitochondria in protein synthesis and mitochondrial regulation of cell signal transduction. The results also suggest that the acupuncture treatment can restore the function of the SR as calcium store during contraction of the muscle and then improve the muscle atrophy.

In this study, the expression levels of GlyRS and MHC-IIb were in line with SFI changes after sciatic nerve injury, but there was slight delay in the recoveries of muscle weight and muscle fiber cross-sectional area. The possible reason for this is that the restoration on the molecular level started earlier than that on the morphological level.

## 5. Conclusion

Peroneus longus muscle atrophy in rats can be improved by EA stimulation of GB30 and ST36 points after sciatic nerve injury induced by penicillin injection. The mechanism of maintaining the normal protein synthesis may be to restore the function of mitochondria and SR and upregulate the GlyRS expression. The significance of this study is to investigate the mechanism of improving muscle atrophy by EA in the perspective of protein synthesis. These findings point to the design of a drug that can upregulate the GlyRS expression and nutrient that can improve recoveries of mitochondria and other organelles. Nevertheless, there are lots of limitations in this research; for example, the upstream and downstream pathways associated with EA and GlyRS expression are not investigated in this experiment, the direct relationship between GlyRS and MHC-IIb is not clear, the downregulation of MHC-IIb is due to protein synthesis reduction, or degradation increase is not clear. These are our future research directions.

## Abbreviations

GlyRS: Glycyl-tRNA synthetase
MHC-IIb: Myosin heavy chain IIb
RT-PCR: Reverse transcriptase-polymerase chain reaction
SD: Sprague-Dawley
SFI: Sciatic functional index
SR: Sarcoplasmic reticulum.
